# Efficient Doxorubicin Loading to Isolated Dexosomes of Immature JAWSII Cells: Formulated and Characterized as the Bionanomaterial

**DOI:** 10.3390/ma13153344

**Published:** 2020-07-27

**Authors:** Esra Cansever Mutlu, Özge Kaya, Matthew Wood, Imre Mager, Kübra Çelik Topkara, Çağrı Çamsarı, Arzu Birinci Yildirim, Ayhan Çetinkaya, Diğdem Acarel, Jale Odabaşı Bağcı

**Affiliations:** 1 Department of Biomedical Engineering, Faculty of Engineering and Architecture, Beykent University, Sarıyer, 34398 Istanbul, Turkey; 2 Scientific Industrial and Technological Application and Research Center, BETUM, Bolu Abant Izzet Baysal University, 14030 Bolu, Turkey; 3 Department of Biology, Faculty of Arts and Sciences, Bolu Abant Izzet Baysal University, 14030 Bolu, Turkey; kaya_o@ibu.edu.tr; 4 Department of Physiology, Anatomy and Genetics, University of Oxford, South Parks Road, Oxford OX1 3QX, UK; matthew.wood@dpag.ox.ac.uk (M.W.); imre.mager@dpag.ox.ac.uk (I.M.); 5 Department of Physiology, Faculty of Medicine, Bolu Abant Izzet Baysal University, 14030 Bolu, Turkey; kubracelik.23@gmail.com (K.Ç.T.); ayhancetinkaya@ibu.edu.tr (A.Ç.); 6 Innovative Food Technologies Development Application and Research Center, Bolu Abant Izzet Baysal University, 14030 Bolu, Turkey; cagri.camsari@gmail.com; 7 Department of Field Crops, Faculty of Agricultural and Environmental Science, 14030 Bolu, Turkey; arzubirinciyildirim@gmail.com; 8 Department of Civil Engineering, Faculty of Engineering and Architecture, Beykent University, Sarıyer, 34398 Istanbul, Turkey; digdemacarel@beykent.edu.tr; 9 Department of Interdisciplinary Neuroscience, Health Sciences Institute, Bolu Abant Izzet Baysal University, 14030 Bolu, Turkey; jalenur@gmail.com

**Keywords:** JAWSII, exosome, doxorubicin, ultrasonication, A549 Cells, natural nanoparticles

## Abstract

Immature dendritic cells (IDc), ‘dexosomes’, are promising natural nanomaterials for cancer diagnose and therapy. Dexosomes were isolated purely from small-scale-up production by using t25-cell-culture flasks. Total RNA was measured as 1.43 ± 0.33 ng/10^6^ cell. Despite the fact that they possessed a surface that is highly abundant in protein, this did not become a significant effect on the DOX loading amount. Ultrasonication was used for doxorubicin (DOX) loading into the IDc dexosomes. In accordance with the literature, three candidate DOX formulations were designed as IC50 values; dExoIII, 1.8 µg/mL, dExoII, 1.2 µg/mL, and dExoI, 0.6 µg/mL, respectively. Formulations were evaluated by MTT test against highly metastatic A549 (CCL-185; ATTC) cell line. Confocal images of unloaded (naïve) were obtained by CellMask^TM^ membrane staining before DOX loading. Although, dexosome membranes were highly durable subsequent to ultrasonication, it was observed that dexosomes could not be stable above 70 °C during the SEM-image analyses. dExoIII displayed sustained release profile. It was found that dynamic light scattering (DLS) and nanoparticle tracking analysis (NTA) results were in good agreement with each other. Zeta potentials of loaded dexosomes have approximately between −15 to −20 mV; and, their sizes are 150 nm even after ultrasonication. IDcJAWSII dexosomes can be able to be utilized as the “BioNanoMaterial” after DOX loading via ultrasonication technique.

## 1. Introduction

Exosomes are natural nanoparticles and have some outstanding features; such as being approximately 40–100 nm in size, and they are composed of different types of varied levels of adhesive proteins and amphiphilic lipid molecules in their structure after the production from various types of cells [1]. Especially, IDc dexosomes have versatile cellular components, such as cell penetration peptides and antigens that can direct the delivery of their cargo (siRNA, miRNA, snRNA, or snoRNA) via both vertical and horizontal transfer [2,3]. Frankly, these naturally occurring events represent all of the cellular traffic which takes place even after modification of the outer surface of exosomes by genetic alteration [4,5]. IDc dexosomes, which do not have a lot of mature specific antigens because they are naïve and fresh structures, have close interaction with surface of cancer cells and can be internalized without causing an immune response [6]. Unlike mature dexosomes, they do not contain any immune system-stimulating molecules. Therefore, they do not trigger any immunogenic rejection from the cancer cells [7,8,9]. Recently, exosome investigations have been shifted mostly to IDc dexosomes due to their therapeutic potential against cancerous cells [10,11]. An abundant amount of exosomes produced from immature dendritic cells can be synthesized in vitro [12]. In addition, they contain favorable cellular components, which the cancer cells prefer [11,13,14,15,16]. According to the literature, mouse primary bone marrow-derived dendritic cells gently isolated from JAWSII (CRL-1194; ATCC) can be used as a pure cell culture that is free from contamination from other types of cells, such as macrophages [17,18].

Synthetic antineoplastic agent doxorubicin, DOX, is an anthracycline derivative. It intercalates into the DNA molecule to function as the killing mechanism of tumor cells. It terminates the progression of the topoisomerase II enzyme by cleaving DNA during transcription. Thereby, it stabilizes the topoisomerase-DNA complex and inhibits the recombination of the DNA double helix [19]. It has been used in the treatment of soft tissue cancers such as lung by both active and passive targeting [5,20,21].

An often encountered limitation is that even small snRNAs cannot be loaded effectively as the desired amount and intact structure. Electroporation is generally used, however, alternative techniques can be applied; including ultrasonication and cellular nanoporation [22]. In the present study, we questioned whether the big anticancer agent doxorubicin can be loaded efficiently to these tiny dexosomes without damaging their structural plasticity, stability, and shape or not. Especially, we focused on one of the most common technique ‘ultrasonication’. With this technique, either DOX unloaded or loaded dexosomes were able to be evaluated as before/after the application of sound energy within the liquid. Dexosome membrane and surface structure were aimed to be evaluated according to a material science perspective. Therefore, NTA and DLS studies were performed at the same time by using same samples. Comprehensively, we intended to put forward whether both dexosome release and cytotoxicity could be performed immediately after reloading of dexosomes; just as lipidomic or polymeric based big molecule loaded bio-nanomaterials.

## 2. Materials and Methods

### 2.1. Pretreated Solutions

In the current study, ExoFreePBS, ExoFreeWater, and ExoFreeFBS were obtained by ultracentrifugation in phosphate-buffered saline (PBS), double-distilled water (ddH_2_O), and fetal bovine serum (FBS). HITACHIMODEL S303922A ultracentrifuge tubes (Hitachi Koki Co., Ltd., Tokyo, Japan) were used following sterilization at 121 °C. As described previously in the literature, ultracentrifugation was carried out overnight at 120,000 g [23,24]. Supernatants were taken gently after ultracentrifugation. DexFreeFBS was kept at −20 °C for further use. DexFreePBS and DexFreeWater were kept at +4 °C [25].

### 2.2. Dexosome Production and Isolation

Immature Dendritic Cell Line, JAWS II, (CRL-1194; ATCC) was purchased from American Type Culture Collection. As described previously, cells were grown at the DMEM-F12 full growth media supplemented with 10% FBS, 4 mM L-glutamine, 1% penicillin-streptomycin, 0.5% amptotecin, and 25 ng/mL murine GM-CSF at 37 °C and 5% CO_2_ environment [16,23]. Cell culture passages were carried out with pouring nonadherent cells directly to a centrifuge tubes; and, adherent cells subjugated pre-treatment with 0.25% trypsin-0.03% EDTA (Gibco) at 37 °C for 10 min. Adherent and non-adherent cells were mixed together to transfer into the new cell culture flasks. Prior to performing dexosome isolation, healthy cells were counted through automated TC20^TM^ cell counter (BioRad, Hercules, CA, USA).

Dexosomes from non-adherent IDc were centrifuged at 300 *g* for 15 min at 4 °C. Then, cell pellets were removed and supernatants were gently poured into 8 mL ultracentrifuge tubes on dry ice. Ultra-centrifugation process was carried out at 17,000 *g* for 20 min at 4 °C to discard all cellular debris. Supernatants were then filtered through TPP^TM^ 0.2 µM sterile filters. Immediately afterwards, ultracentrifugation was performed at 120,000 *g* for 60 min at 4 °C. Dexosome enriched pellets were resuspended three times in ExoFree PBS as (3 × 50 µL) for maximum exosome retrieval within sterile cryotubes. Cryotubes were kept at −80 °C for further exosome analysis [16].

### 2.3. Evaluation of Dexosomal RNA and Protein Content

Dexosomal total RNA content was determined using two column base commercial RNA isolation kits (innuPREP RNA Minianalytik^TM^; Jena, Germany). Likewise, miRNA was also extracted by (Omega Bio-tek, Inc., Guangzhou, China). Samples were first suspended in 50 µL of DexFree PBS and mixed with lysis buffer provided in each kit. Isolated RNA molecules were eluted from the columns via centrifuging in nuclease-free water and total RNA and miRNA quantification measurements were carried out via QuantiFluor^®^ RNA Dye on fluorometry (Quantus^TM^ Fluorometer, Promega, Madisson, WI, USA; Thermo Scientific/2000^TM^ Nanodrop, Wilmington, DE, USA). Control samples contained only nuclease free water. In order to prevent RNase contamination, the working space and all apparatus, such as pipette tips, centrifuge tubes were cleaned with RNase-ExitusPlus^TM^.

Total protein content of the JAWS II dexosome samples suspended in DexFreePBS were evaluated with Bradford (1976) [26] method through using Pierce^TM^ Coomassie [27] Protein Assay Kit. The amount of total protein was measured at 595 nm, by using a spectrophotometer (Jasco V-530 UV–vis spectrophotometer, Jasco International Corporation, Tokyo, Japan) and quantified as bovine serum albumin equivalent [28] value.

### 2.4. Confocal Studies after CellMask Membrane Staining

CellMask^TM^ Green Plasma Membrane Stain was used as the Confocal Image probe to evaluate the membrane images of dexosomes following isolation at 488 nm and low and high magnification. 3 µg/mL volume of stain was used as previously recommended by El-Andaloussi et al. [16]. Stain and dexosomes were mixed 1:1 (v/v) and incubated 30 min at 37 °C. Then it was centrifuged at RT for 10 min at 4000 *g* maximum by using Amicon Ultra-2 filter. It was recovered as a concentrated solute by inverting the Amicon Ultra-2 filter device and concentrate collection tube in order to discard excess dye with ExoFreeWater by reverse spin at RT for 2 min at 1000 *g*. Thereby, the sample was transferred to the tube of the Amicon Ultra-2 filter device from the filtrate collection tube.

### 2.5. Loading of Dexosomes with Doxorubicin by Ultrasonication

Doxorubicin was kindly donated for the present study from DEVARGE. Based on the previously published data, three different IC50 values of DOX as 1.8 µg/mL, 1.2 µg/mL and 0.6 µg/mL were evaluated for loading into dexosomes [29]. Ultrasonication mixture was obtained by mixing dexosome and doxorubicin in 1:1 (v/v) in ExoFreePBS. Ultrasonication procedure were performed by using Sonic Vibra Cell Sonics and Materials^TM^ with CV1 6089 Tip. As previously described, ultrasonication was performed as 20% amplitude, two cycles of 20 s on/off with 5 min cooling period between each cycle in order to obtain high DOX loading performance [30,31]. Samples were kept in dry ice during ultrasonication procedure (Supplementary Materials Video S1 and Video S2). Excess DOX amount was calculated from the dExoIII solution during drug release studies within 30 min.

### 2.6. NTA Analysis

Nanoparticle tracking analysis was performed by using the Nanosight NS500 instrument (Malvern Panalytical, Malvern, UK) and the NanoSight NTA 3.1 software. The samples were diluted to reach the linear detection range of the device (5–15 × 10^8^ articles/mL) and three 30 s videos were recorded at the cCMOS camera type camera level 14. The videos were analyzed using the default auto settings of the software.

### 2.7. DLS Analysis

Dexosome solution was diluted with 2 mL ExoFree PBS and measurements were performed in order to detect size and stability by Malvern nanosizer/zetasizer nano-ZS ZEN 3600, respectively. While the size measurements were performed by using dip cell cuvettes; for zeta disposable sizing, cuvettes were used.

### 2.8. SEM Analysis

Diluted stock exosomes 2 mL dropped onto metal grids with double sided adhesive carbon tape by using sterile Pasteur pipettes. After drying, it was coated with gold to ~500 × 10^−8^ cm in thickness using sputter coater under high vacuum, 0.1 Torr, 1.2 kV at 27 °C ± 1 °C. The surface morphologies of coated samples were evaluated by using scanning electron microscopy [32].

### 2.9. In Vitro Cytotoxic Assay

MTT test was performed to evaluate cytotoxicity. Highly metastatic A549 cells (CCL-185, American Type Culture Collection, Manassas, VA, USA) was obtained from stocks of Bolu Abant Izzet Baysal University, Department of Physiology. Cells were cultured in DMEM F-12 media supplemented with 10% FBS, 1% penicilin streptomycin, and 0.5% amptotecin. 3 mL trypsin EDTA was used to detach the cells. Then, 96-well plates were seeded with cells, each one contained 5 × 10^3^ cells. DOX loaded dexosomes were administered as 0.6, 1.2, and 1.8 µg/mL with a previous set dilution ratio. For comparison, three IC50 DOX dosage 0.6, 1.2, and 1.8 µg/mL were tested together [16]. Each dose was repeated three times. Empty dexosomes, water, PBS, and DMSO were used as the control group.

All the experiments were performed in triplicate. The data were calculated and demonstrated as mean ± SD of three independent experiments. * *p* < 0.05 and ** *p* < 0.005 compared to control and other treatments were considered statistically significant. ANOVA (one-way analysis of variance) were performed for cell viability test and followed by Duncan’s multiple range tests using SPSS 18 version (SPSS Inc., Chicago, IL, USA).

### 2.10. Drug Release Analysis

Drug release experiments were performed onset by DOX calibration curve ranging 0 to 3.6 µg/mL in PBS solution (R^2^ = 0.958). DOX release profile obtained from 1.8 µg/mL DOX loaded dexosomes. The dexosome nanoformulation sample (2 mL) was put into a Slide-A-Lyzer MINI dialysis microtube cutoff of 3500 Da (Pierce, Rockford, IL, USA) and dialyzed in falcon tubes against 45 mL of PBS buffer at 37 °C within shaker at 90 rpm. Following the renewal of PBS, the dialysis medium was divided in 3 mL volume aliquots obtained at 0.5, 1, 2, 3, 4, 6, 12, 24, 48, and 72 h intervals. The amount of free DOX in each aliquot from dialysis medium was determined by measuring specific emission peak intensities of DOX at 480 nm using a fluorescence and absorbance spectrophotometer. The drug release percentage was plotted against time. 3 mL of the remaining suspensions in the microtubes were diluted with acetonitrile in a ratio of 1:1 (v/v) to disassemble the DOX-loaded dexosomes. The DOX release profile of dexosomes over 72 h was calculated by using the equation obtained from the DOX calibration curve.

## 3. Results

### 3.1. Evaluation of Dexosomal RNA and Protein Content

Bradford (1976) method was used to determine the exosomal protein concentration. 31.9958 ± 5.8310 µg/10^6^ cells in BSA (bovine serum albumin) medium were detected from 5.25 × 10^9^ JawsII naïve dexosomes. This proved highly abundant protein content on the surface of dexosomes. No protein was detected in the control sample (DexFreePBS) [29]. No miRNA was detected. Total RNA was 1.43 ± 0.33 ng/10^6^ cell from JAWS II dexosomes. During fluorometric measurements, total RNA was not detected in the control samples.

### 3.2. Confocal Studies after CellMask Membrane Staining

**Figure 1:**
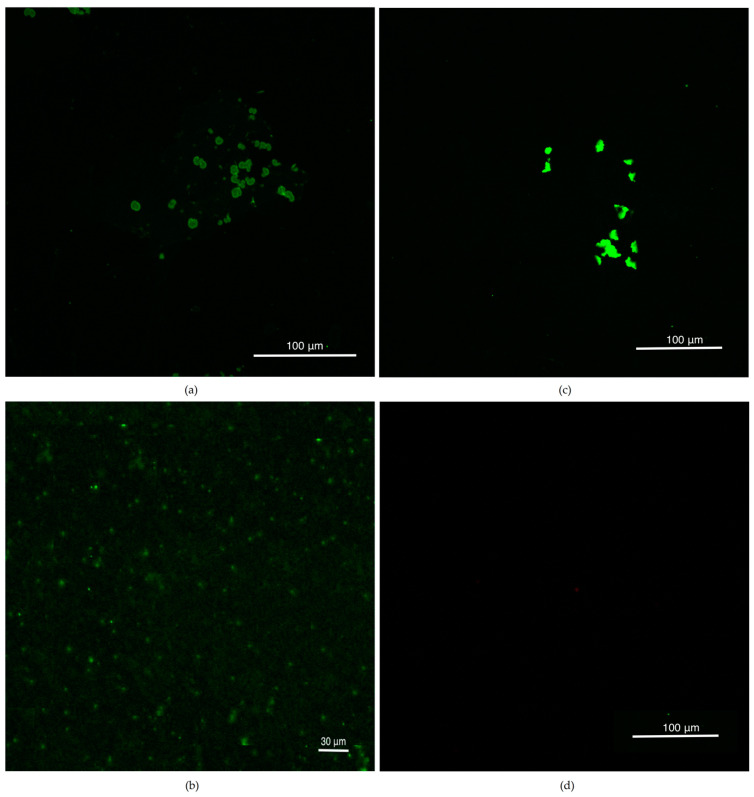
Confocal images at 488 nm with several magnifications as defined while (**a**) high and (**b**) low magnification of naïve dexosomes 2 h following staining (**c**) only CellMask Green dye (**d**) blank as the control.

Confocal Studies were performed by using CellMask Plasma Membrane Stain^TM^. Membrane stability of naïve dexosomes were tested before drug loading. Dexosome images were compared with both blank control and CellMask Green Dye under Nikon^TM^ confocal microscope (Figure 1). Stained dexosomes could be visible and tend to highly dense agglomeration in water dilution during the analyses. Membrane structures were not observed as being fully rigid and spherical; on the contrary, sliding and dynamic rounded structures were observed.

### 3.3. NTA Analysis

**Figure 2:**
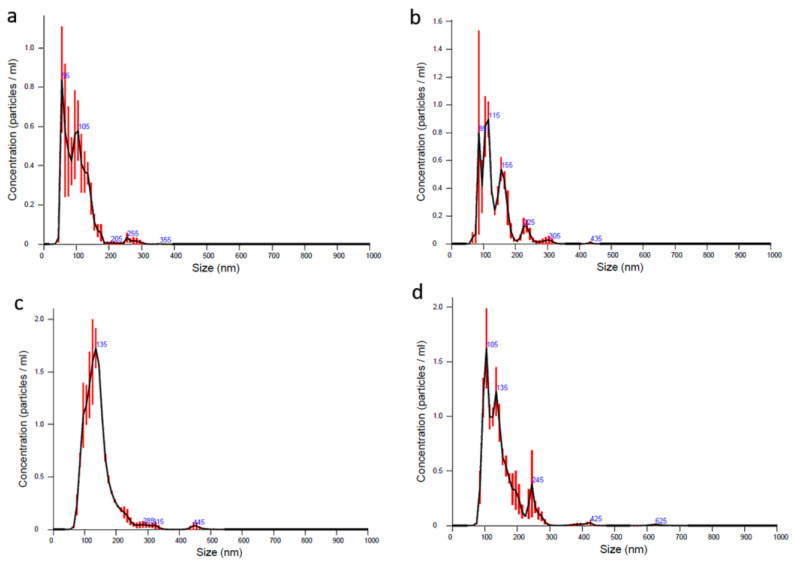
NTA results of dexosomes (**a**) naive: 99 nm; (**b**) dExOI: 134 nm; (**c**) dExOII: 142 nm; (**d**) dExOIII: 148 nm.

Ultrasonication was an effective method for synthetic drug loading as previously described by Kim et al. and the other literatures [29,30,31]. All dexosome concentration after isolation was measured approximately as 1.4 × 10^9^ particles/mL for each cryotube of dExOI, dExOII, and dExOIII. Nanoparticle tracking analyses were performed at ¼ dilution of ExoFreePBS for dExOI, dExOII, dExOIII, and control ExoFreePBS. While naïve particles were 99 nm; dExOI, dExOII, and dExOIII were 134, 142, and 148 nm mean diameter in size, respectively. The increase in drug concentration resulted in large dexosome sizes. Likewise, small fractions of naïve dexosomes were above 200 nm were present in collected samples, even after their production (Figure 2).

### 3.4. DLS Analysis

**Figure 3:**
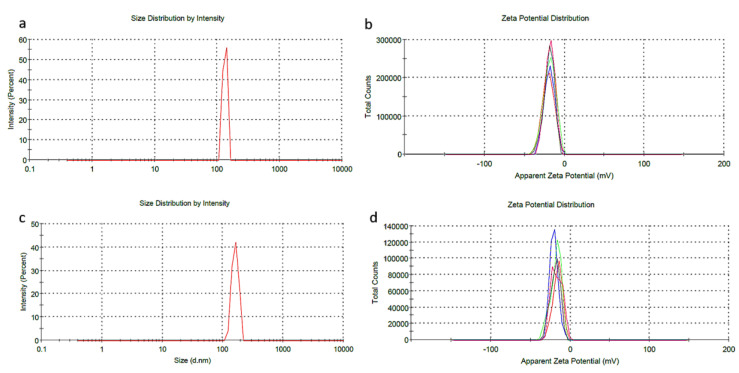
DLS results of (**a**) dexOI are 133 nm and (**b**) −20 mV; (**c**) dExOIIII are 161 nm and (**d**) −15 mV.

Dynamic light scattering was performed in order to determine size vs. stability (according to surface charge) by zeta-sizer measurements of dExOI, dExOII, and dExOIII. Monodispersed size distribution was obtained for each DOX loaded dExO exosomes and naïve samples. Size was increasing by loading DOX amount to dEXO (Figure 3a,c). On the other hand, measurements showed that ultrasonication is one of the best synthetic drug loading methods; so that, zeta potentials were between −10 to +20 mV. Moreover, data showed that dexosome size and capacity increased in diameter by DOX loading; however, zeta measurements displayed a small decrease in the surface charge of dexosomes (Figure 3b,d). Neither the destability or disintegration have been detected for the three nanoformulations.

Results showed that IDc dexosomes (dExO) were highly small ≤ 99 nm structures with a surface charge of −10.7 ± 7.39 mv before drug loading. Z-intensity measurement did approximately results overlap with NTA measurements when it was taken into account their standard errors. NTA results, using Brownian motion rate measurements of particles statistically, were shown in Figures S1–S4. Size diameters obtained from NTA summarized more accurate results than Z-intensity as displayed in Table 1.

**Figure 4:**
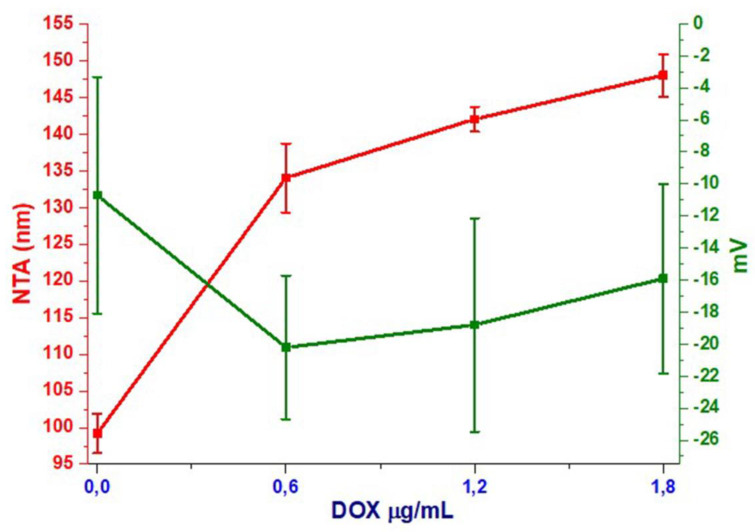
Changes in size and zeta potential of dexosomes based on the amount of DOX in dExO.

Surface charge of naïve dexosomes were measured at −10.7 ± 7.39 mV. It increased significantly after drug loading; such as, dExOI −20.2 mV in Figure 4; which did not overlap with the results of the study by Kim et al. [29]. In their study, they performed paclitaxel loading to macrophage exosomes via ultrasonication. They reported that the surface charge of their exosomes did not show remarkable change before and after paclitaxel loading. On the contrary, our results displayed remarkable change in surface charges after ultrasonication (Figures S5–S8).

### 3.5. SEM Analysis

**Figure 5:**
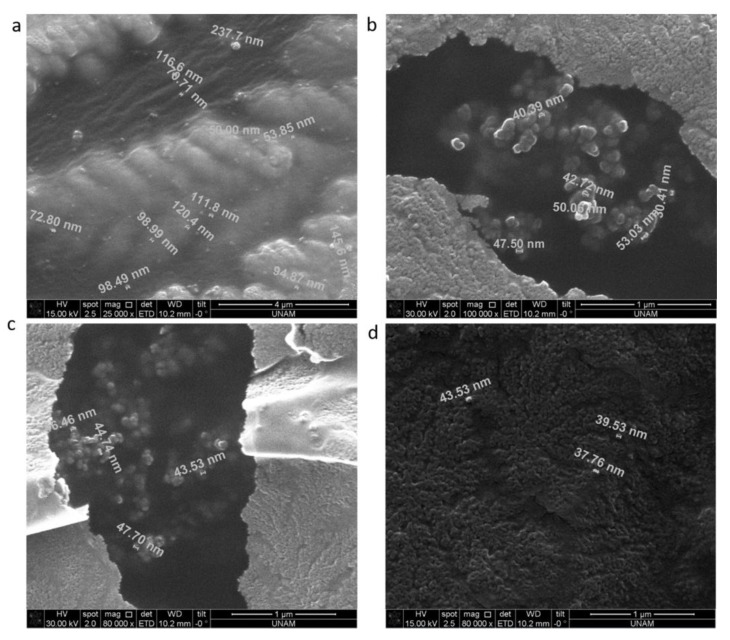
SEM images of dexosomes (**a**) naive; (**b**) dExOI; (**c**) dExOII; (**d**) dExOIII, respectively.

Fixing dexosomes to the carbon tapes was a complicated procedure. Dexosomes were diluted at 40× in ExoFreeWater inside the 2 mL cryo-tube. After pipetting three drops onto carbon tape, they were placed in room temperature in order to observe their durability. Obtaining just one dexosome image was possible with dilution. After Au coating, SEM measurements were performed. Dexosome spherical structures were denatured with increased magnification and temperature. Above 75 °C temperature, dexosomes were not robust. While a dexosome was vibrating; dexosomes in clusters tended to agglomeration, Figure 5 surface morphology of naïve, dExOI, dExOII, and dExOIII dexosomes are almost uniform. No impurity was noticed during SEM image study. Additionally, SEM images proved that the rising DOX amount in dExO increases size without deformation at the spherical structure.

### 3.6. In Vitro Cytotoxic Assay

**Figure 6:**
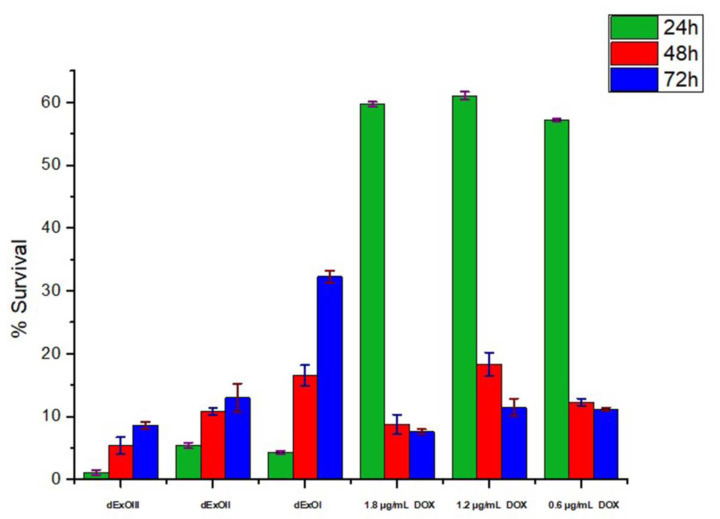
Cytotoxicity of dExO formulations and DOX dosages against to A549 cancer cell line.

MTT assay was applied in order to evaluate the cytotoxicity of DOX loaded dexosomes (dExO). Thereby, highly metastatic and resistant A549 (CCL-185) human lung cancer cell line was selected. Three IC50 DOX concentrations as (0.6; 1.2; and 1.8 µg/mL) were tested as naïve and dExOI, dExOII, and dExOIII respectively. Besides, these three IC50 doses were evaluated within same 96-well plate with controls. Control group has been designated as ExoFreePBS, ExoFreeWater, DMSO, and naïve dexosomes. There was effective toxicity of three dExO formulations against to A549 cell line within 72 h. Survival rate was calculated as 9% as minimum of dExOIII. The cell survival calculated as 13% of dExOII and 32% of dExOI formulations at the end of 72 h. Utmost each DOX dose shows cytotoxicity below IC50 value as previously reported in the literature (Figure 6) [29].

**Figure 7:**
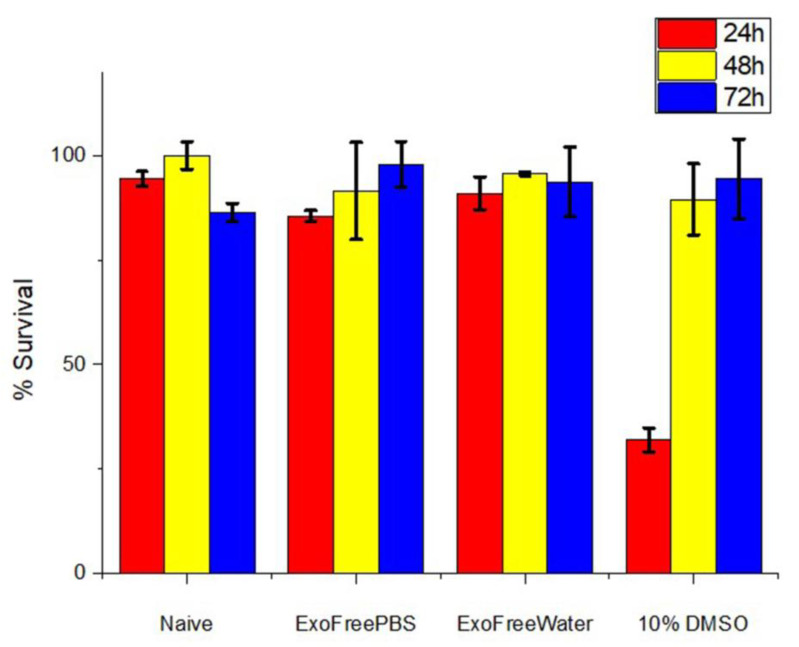
Survival rate of control groups; naïve dexosomes, PBS, water, and DMSO against A549 cancer cell line.

MTT results showed DOX loaded dexosomes (dExOI, dExOII, and dExOIII) against to A549 cell line had more cytotoxic effect than DOX dosages administered alone. Control groups demonstrated that A549 cell line was highly metastatic. Survival rate in DMSO was 32% after 24 h which increased to 95% even after 72 h. Figure 7 naive dexosomes had a survival rate of approximately 93% at the end of 72 h. These results suggested that DOX loaded dexosomes (dExOI, dExOII, and dExOIII) are highly favorable cytotoxic bionanomaterial against to A549 cancer cells.

### 3.7. Drug Release Analysis

It was performed by using concentration values of the peak intensities from standard calibration curve plotted against time. The DOX release percentage (w/w) versus time profile of dExOIII showed that no release happened for the first half an hour. This proved a successful DOX loading during ultrasonication. After 72 h, dexosome samples were disassembled by acetonitrile to obtain a loaded and retained DOX amount.

**Figure 8:**
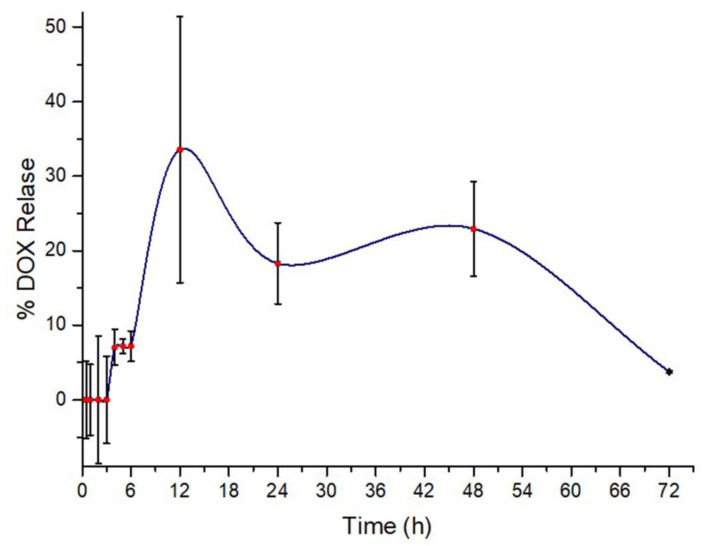
Drug release profile of dExOIII during 72 h.

Drug release studies performed simply by using DOX/UV curve. No DOX was released from the dExOIII particles until the end of third hour. After 4, 5, 6 h, dexosomes released at the same amount; 7% of total DOX. Surprisingly, 34%, 18%, and 22% DOX release were measured following 12, 24, 48 h, respectively. 3% DOX was retained even at the end of 72 h, as displayed in Figure 8.

## 4. Discussion

In the present study, dexosomes (dExO) from JAWSII (ATCC^®^ CRL-11904^TM^) immature dendritic cells (IDc) were isolated and their synthetic antineoplastic drug DOX loading potential were evaluated through ultrasonication. By this investigation, we revealed more about the handling of IDc dexosomes as a promising nanoformulation from a nano-material perspective without genetic change of surface proteins [5].

After dexosomes were isolated, a small amount of total RNA would have been obtained; however, dexosomal miRNA was not detected. This should be investigated further in detail; because dexosomes cannot be loaded with drug unless there is enough space in them. Even exogenous loading of small mRNA molecules into EVs is a complicated challenge which needs to be overcome [22]. This technique represents loading big molecules, rather than encapsulation, as described by Srivastava, A. et al. and it differs from the others by this aspect [33]. Therefore, cell culture and drug release studies proved that DOX amount could be loaded successfully and molecular function was not diminished in the ultrasonication process. Surface charges of dExO formulations after loading decreased partially; consequently, DOX increased the stability of dexosomes. Protein content was measured as 31.83 µg/mL for each cryo dexosome stock content. This did not affect drug loading amount and confocal image studies as an impurity. Membrane structures of dexosomes were observed under confocal microscope as being highly pure and stable for the biggest dexosomes. They tended to dynamic motion under high and low magnification. This should be investigated further just as ascribed status of exosomes reported by Cvjetkovic et al. [34]. Lowest size ones were captured by SEM images without statistical calculation; meanwhile, all dexosome size distribution conveniently were able to measure by NTA.

In vitro study was performed with a highly metastatic A549 lung cancer cell line; so that 20% DMSO was able to kill metastatic cells as control group following 24 h and 48 h administration. However, naïve dexosomes, ExoFreePBS, and ExoFreeWater have a high survival rate. dExO nanoformulations have better cytotoxicity than DOX itself. Even if a small amount of apoptotic bodies and extracellular vehicles were detected by NTA, they did not affect drug loading capacity and cytotoxicity performance of dExO.

The smart DOX release profile has been noticed. DOX gradually released from dExOIII firstly followed a rising trend. Then, it exhibited a decreasing trend over 72 h of measurement. DOX has reached optimum peak (total 55% of DOX) at 12 h and 3% DOX has been retained, even after 72 h. Release percentage displays sustained release profile just as described in Agrawal et al. They showed the paclitaxel-loaded exosomes (ExoPAC) had sustained a release profile up to 48 h in vitro using PBS (pH 6.8) [35].

Today, investigations about nanoformulations tend to evolve as potential exosomal studies for diagnosis and therapy [36,37,38,39,40,41,42,43]. Especially, Yang et al. [19] have recently used ultrasonication and their results suggested that DOX loaded tumor-cell-exocytosed exosome-biomimetic porous silicon nanoparticles (PSiNPs) have a clinical potential for cancer treatments [10].

The one of well-known challenges have being considered as loading desired molecule cargo into them and the delivery of this cargo to the site of action. Based on the previously published data, confocal microscopy alone are not sufficient to evaluate the roles of the biological materials [44]. In this study, isolated dexosomes as the natural nanoparticles were evaluated according to their drug loading potential with synthetic antineoplastic agent DOX. Even if ultrasonication technique has been applied before in the literature; this study firstly showed their size and stability would be able to be interpreted comprehensively by using both NTA and DLS results before/after DOX loading [16,29]. DLS results were thought represented agglomeration property of dexosomes. When the standart errors of DLS results were taken into account, they are overlapped with NTA results. From this perspective, NTA and DLS were good agreement with each other. Further investigation should be developed according to pharmaceutical perspective and it should be designed as in situ, preclinical, and clinical studies. It is needed to perform an evaluation on the effect on cell cycle and apoptosis as described in the literature [12,45].

## 5. Conclusions

The results of the current study suggested that immature dendritic cells of JAWSII (CRL-1194 ATCC) could be used as a promising biomedical nanomaterial model without any genetic alteration. In the present study, fate of total RNA and protein content of dexosomes were not evaluated in detail. DOX-loaded dexosomes have been characterized according to both DOX release and in vitro cytotoxicity tests against highly metastatic A549 lung cancer cell line. Consequently, more cancer and healthy cell lines should be investigated as the response against to these candidate bionanomaterial. Since miRNA contents were not detected, from a material science perspective, further NMR and DSC investigation will be needed to focus on stability and chemical structures of several types of dexosomal RNA throughout the ultrasonication process.

## Figures and Tables

**Table 1:** Comparative NTA, Z-intensity, and surface charge results of naïve and dExO dexosomes.

Sample/Method	Size by NTA (nm)	Z-Intensity (nm)	Zeta Potential (mv)
Naïve	99.2 ± 2.7	209.4 ± 81.7	−10.7 ± 7.39
dExOIII	148 ± 2.9	161.2 ± 19.26	−15.9 ± 5.91
dExOII	142 ± 1.7	162.5 ± 17.14	−18.8 ± 6.67
dExOI	134 ± 4.7	133.2 ± 9.6	−20.2 ± 4.48

## Supplementary Materials

The following are available online at https://www.mdpi.com/1996-1944/13/15/3344/s1, Video S1 and S2: Ultrasonication process to dexosomes; Figures S1–S4: NTA results of dexosomes; Figures S5–S8: Zeta potential results of dexosomes.
