# Supplementary material for: Efficient Doxorubicin Loading to Isolated Dexosomes of Immature JAWSII Cells: Formulated and Characterized as the Bionanomaterial
Source: Materials (Basel). 2020 Jul 27;13(15):3344. doi: 10.3390/ma13153344 (PMC7435586; doi:10.3390/ma13153344)
Supplement: Supplementary file 1 [file materials-13-03344-s001.zip › materials-856243-supplementary.docx]

Supplementary Materials

Efficient Doxorubicin Loading to Isolated Dexosomes of Immature JAWSII Cells: Formulated and Characterized as the Bionanomaterial

Esra Cansever Mutlu ^1,2,^*, Özge Kaya ^3^, Matthew Wood ^4^, Imre Mager ^4^, Kübra Çelik Topkara ^5^, Çağrı Çamsarı ^6^, Arzu Birinci Yildirim ^7^, Ayhan Çetinkaya ^5^, Diğdem Acarel ^8^ and Jale Odabaşı Bağcı ^9^

^1^ Department of Biomedical Engineering, Faculty of Engineering and Architecture, Beykent University, Sarıyer, 34398 Istanbul, Turkey

^2^ Scientific Industrial and Technological Application and Research Center, BETUM, Bolu Abant Izzet Baysal University, 14030 Bolu, Turkey

^3^ Department of Biology, Faculty of Arts and Sciences, Bolu Abant Izzet Baysal University, 14030 Bolu, Turkey; [kaya_o@ibu.edu.tr](mailto:kaya_o@ibu.edu.tr)

^4^ Department of Physiology, Anatomy and Genetics, University of Oxford, South Parks Road, Oxford OX1 3QX, UK; [matthew.wood@dpag.ox.ac.uk](mailto:matthew.wood@dpag.ox.ac.uk) (M.W.); [imre.mager@dpag.ox.ac.uk](mailto:imre.mager@dpag.ox.ac.uk) (I.M.)

^5^ Department of Physiology, Faculty of Medicine, Bolu Abant Izzet Baysal University, 14030 Bolu, Turkey; [kubracelik.23@gmail.com](mailto:kubracelik.23@gmail.com) (K.Ç.T.); [ayhancetinkaya@ibu.edu.tr](mailto:ayhancetinkaya@ibu.edu.tr) (A.Ç.)

^6^ Innovative Food Technologies Development Application and Research Center, Bolu Abant Izzet Baysal University, 14030 Bolu, Turkey; [cagri.camsari@gmail.com](mailto:cagri.camsari@gmail.com)

^7^ Department of Field Crops, Faculty of Agricultural and Environmental Science, 14030 Bolu, Turkey; [arzubirinciyildirim@gmail.com](mailto:arzubirinciyildirim@gmail.com)

^8^ Department of Civil Engineering, Faculty of Engineering and Architecture, Beykent University, Sarıyer, 34398 Istanbul, Turkey; [digdemacarel@beykent.edu.tr](mailto:digdemacarel@beykent.edu.tr)

^9^ Department of Interdisciplinary Neuroscience, Health Sciences Institute, Bolu Abant Izzet Baysal University, 14030 Bolu, Turkey; [jalenur@gmail.com](mailto:jalenur@gmail.com)

* Correspondence: esramutlu@beykent.edu.tr

**Figure S1.** NTA result of Naïve dexosomes.

**Figure S2.** NTA result of dExoI.

**Figure S3.** NTA result of dExoII.

**Figure S4.** NTA result of dExoIII.

**Figure S5.** Zeta potential of Naive dexosomes.

**Figure S6.** Zeta potential of dExoI.

**Figure S7.** Zeta potential of dExoII.

**Figure S8.** Zeta potential of dExoIII.

**Video S1 and S2:** Ultrasonication process to dexosomes.
